# The motivational climate perceived by young soccer players regarding their coaches, parents, and peers on sport optimal functioning: a cluster analysis

**DOI:** 10.3389/fpsyg.2025.1564391

**Published:** 2025-07-25

**Authors:** Natalia Martínez-González, Francisco L. Atienza-González, Lorena González-García, Isabel Balaguer

**Affiliations:** ^1^Faculty of Psychology and Speech Therapy, University of Malaga, Málaga, Spain; ^2^Faculty of Psychology and Speech Therapy, University of Valencia, Valencia, Spain

**Keywords:** self-determination theory, achievement goal theory, young athletes, significant others, motivational climate

## Abstract

The aim of this study was (a) to describe the perceived motivational climate profiles created by coaches, parents, and peers of young football players, and (b) to analyse the implications of these profiles on goal orientations, motivation, psychological needs, and indicators of well- and ill-being. The participants were 876 football players (*M* = 13.57; *SD* = 1.17) who completed a multi-section questionnaire at the beginning of the sports season. Cluster analyses identified four profiles (*empowering, disempowering, high mixed, low mixed*) based on youth perceptions of empowering and disempowering climates created by coaches, parents, and peers. Multivariate analyses, controlling for gender and age, revealed that those young players categorized in the most adaptive profiles (high empowering climates and low disempowering climates) demonstrated optimal functioning, including task orientation, higher autonomous motivation, need satisfaction, and vitality. Conversely, those in the most maladaptive profile (low empowering climates and high disempowering climates) experienced the worst consequences (high levels of ego orientation, controlled motivation, no motivation, need frustration, physical and emotional exhaustion). Regarding mixed profiles, although the analyses reveal that combining empowering and disempowering behaviors limits the benefits of empowering climates, and low involvement hinders athletes’ development, future explorations are necessary to better interpret these profiles. As a whole, the results highlight the importance of considering the combined roles of coaches, parents, and peers, and suggest that interventions aimed at fostering empowering behaviors and reducing disempowering ones across these social agents may help support youth athletes’ optimal functioning.

## 1 Introduction

Several psychosocial theories postulate, that both the behaviors and interaction patterns adopted by significant others within the sports context exert a determining influence—either positive or negative—on sport participants. Contemporary theories of motivation, specifically Self-determination theory (SDT) (Deci and Ryan, 2000) and Achievement Goal Theory (AGT) (Ames, 1992; Nicholls, 1989), have emphasized the important role played by the social environment, represented mainly by significant others (parents, peers, and coaches) in the sporting experience of athletes and associated outcomes. The environment that surrounds them, represented in these motivational theories by the motivational climate, has been widely studied for its substantial impact on how athletes feel, think, and behave, with important implications for their health and engagement in sports (Appleton and Duda, 2016; Duda, 2013). While research has focused primarily on coaches, there is growing recognition of the complementary roles parents and peers play in shaping social environments that can foster or hinder young athletes’ well-being, enjoyment, and optimal functioning (Fredricks and Eccles, 2004; Harwood and Knight, 2015; Le Bars et al., 2009; O’Rourke et al., 2014).

Within the framework of SDT, emphasis is placed on the social environment’s ability to either satisfy or frustrate the basic psychological needs for competence, autonomy, and relatedness (Deci and Ryan, 1985, 2000; Ryan and Deci, 2000, 2017). These three needs, considered innate and universal, are described as “psychological nutrients” (Ryan, 1995, p. 410) that promote well-being and motivation. In sports, when the social environment fosters athletes’ autonomy, competence, and relatedness, it is more likely that they will experience higher-quality motivation (i.e., autonomous motivation) in sports practice and enhanced well-being (Balaguer et al., 2011b; Balaguer et al., 2012; Fabra et al., 2023; González et al., 2017). Conversely, when these needs are thwarted, athletes are more likely to exhibit lower-quality motivation (i.e., controlled motivation) or even amotivation, alongside a decline in optimal functioning during sports practice (Balaguer et al., 2012; Bartholomew et al., 2011; Castillo et al., 2012; Fabra et al., 2023; González et al., 2017).

On the other hand, AGT focuses on how significant others convey their perceptions of success and competence through motivational climates, distinguishing between task-involving and ego-involving climates. Task-involving climates encourage athletes to evaluate their competence based on self-referenced criteria, emphasizing effort, learning, and mastery of tasks. In these environments, mistakes are seen as a natural part of the learning process, and cooperation and individual contributions are valued (Nicholls, 1989; Duda, 2001). Such climates promote team cohesion (García-Calvo et al., 2014) as well as individual well-being [see systematic review by Harwood et al. (2015)]. In contrast, ego-involving climates lead athletes to assess their competence through normative comparisons, fostering rivalry and placing focus on external outcomes rather than the learning process. In such contexts, mistakes are often punished, and reinforcement is typically directed toward athletes who demonstrate superiority over others (Newton et al., 2000; Reinboth and Duda, 2006). As a result, athletes in these climates tend to experience distress and may develop a desire to abandon sports participation (Fabra et al., 2021; Sarrazin et al., 2002).

To integrate the principles and theoretical concepts of both SDT and AGT, the integrated model of empowering and disempowering climates was recently developed (Duda, 2013; Duda et al., 2024). This model combines the dimensions proposed by the two theories, offering a broader perspective on motivational climates while detailing the psychosocial characteristics that predominate within these climates and their potential consequences (Balaguer et al., 2021). The result is a hierarchical model that describes motivational climates as a function of whether they are more or less empowering or more or less disempowering. When the motivational climate is empowering, it is characterized by high task-involving, autonomy supportive, and social supportive behaviors. In contrast, a disempowering climate includes high degrees of ego-involving and controlling behaviors (Duda, 2013; Duda and Appleton, 2016; Duda et al., 2024).

Despite the undeniable influence that significant others, such as parents and peers, can exert—particularly in the context of youth sports—this model has primarily focused on analysing the motivational climates created by coaches since its inception, as reviewed by Birr et al. (2023). In this regard, previous research has demonstrated that empowering climates created by coaches are associated with athletes’ optimal functioning and/or greater intentions to continue participating in sports (Castillo-Jiménez et al., 2017; Krommidas et al., 2016; Martínez-González et al., 2021; Mosqueda et al., 2019; Ruiz et al., 2021). These positive outcomes can be attributed to the capacity of empowering climates to satisfy the three basic psychological needs (Chu et al., 2021; Mosqueda et al., 2022), while also promoting task orientation (Duda et al., 2018; Duda et al., 2024; Ruiz et al., 2021), autonomous motivation (Fenton et al., 2017; Mosqueda and López-Walle, 2022), and athletes’ well-being (Appleton and Duda, 2016; Hancox et al., 2017; Krommidas et al., 2016; Krommidas et al., 2022).

In contrast, disempowering climates created by coaches have been associated with compromised functioning and a higher likelihood of dropout intentions (Castillo-Jiménez et al., 2022; Krommidas et al., 2016). These negative consequences can be attributed to the tendency of disempowering climates to thwart basic psychological needs (Chu et al., 2021; Mosqueda et al., 2022; Mosqueda and López-Walle, 2022), foster an ego-oriented goal perspective (Duda et al., 2018; Duda et al., 2024; Ruiz et al., 2021), and promote controlled motivation (Mosqueda and López-Walle, 2022; Ruiz et al., 2021) alongside ill-being (Hancox et al., 2017; Into et al., 2020).

However, what about the empowering and disempowering climates created by parents or peers? The existing literature has largely overlooked the roles of both groups as key social agents in youth sports. This constitutes a limitation in the study of motivational climates in sports, as there is currently a lack of data of parental and peers’ motivational climates within the empowering and disempowering framework. As a result, this gap limits the potential for a deeper understanding of their implications for the sporting experiences of young athletes.

Regarding the role of parents, it should not be forgotten that, during childhood and adolescence, parents continue to be the primary social agents shaping young athletes’ experiences and participation in sports (Fredricks and Eccles, 2004; Harwood and Knight, 2015). In fact, they provide essential material, emotional, organizational, and financial support, which serves as the foundation for enabling children to participate in sports (Holt and Knight, 2014; Wolfenden and Holt, 2005). It seems that in some variables, such as motivation, parents’ influence on young athletes has surpassed that of coaches (Amorose et al., 2016; Atkins et al., 2015; O’Rourke et al., 2014). This assumption seems to have been confirmed recently by Gao et al. (2024), who concluded in a systematic review that “parents play both unique and synergistic multidimensional roles in motivating young athletes” (p. 1).

From the perspective of SDT, several studies have shown that young athletes’ perception of parents who support their autonomy is linked to the satisfaction of basic psychological needs (Gaudreau et al., 2016) and greater autonomous motivation (Amorose et al., 2016; Gagné, 2003; O’Neil and Amorose, 2021). However, the perception of controlling parenting styles has been associated with controlled motivation or lack of motivation, and negative emotions (see Gao et al., 2024). Also, from the framework of AGT, the literature highlights how the creation of motivational climates by parents that promote learning and effort is positively associated with their children’s task orientation (Waldron and Krane, 2005; Weltevreden et al., 2018), autonomous motivation (O’Rourke et al., 2014), enjoyment (Sánchez-Miguel et al., 2013; Atkins et al., 2015; Williams et al., 2022) and intention to continue playing sport (Atkins et al., 2015; Williams et al., 2022). In contrast, the creation of ego-involving motivational climates by parents is positively associated with their children’s ego orientation (Waldron and Krane, 2005; Weltevreden et al., 2018), extrinsic motivation (O’Rourke et al., 2013), fear of failure (Coutinho et al., 2024), and less enjoyment (Atkins et al., 2015).

The case of motivational climates created by peers has generated some interest in the sports domain, often analyzed alongside and compared to the motivational climate created by coaches. Peers, particularly during adolescence, play a crucial role in shaping the quality of the sporting experience, significantly contributing to whether it is enjoyable or unpleasant (Jowett and Lavalee, 2007). From the perspective of SDT, studies suggest that peers may have a greater influence than coaches in satisfying the basic psychological needs for competence and relatedness [see the review by Chu and Zhang (2019)]. Furthermore, perceived autonomy support from peers has been associated with higher autonomous motivation among youth athletes (Ramis et al., 2013). In studies framed within AGT, findings indicate that a task-involving climate created by peers is positively linked to the satisfaction of basic psychological needs (Jõesaar et al., 2011), intrinsic motivation (Jõesaar et al., 2011, 2012), as well as team cohesion and athlete satisfaction (García-Calvo et al., 2014). Additionally, such climates are associated with stronger intentions to continue participating in sports (Atkins et al., 2015; Le Bars et al., 2009).

Given the above, two key aspects emerge: the importance of jointly studying the motivational climates created by coaches, parents, and peers in youth sports, and the notable scarcity of studies addressing this from the perspective of the integrated model of empowering and disempowering motivational climates, which combines the principles of SDT and AGT to provide a more comprehensive understanding (Duda et al., 2024). To address these gaps in the literature, athletes’ perceptions of the motivational climates created by these social agents will be analyzed together using a person-centered approach, which can reveal meaningful patterns in athletes’ experiences and shed light on how combinations of empowering and disempowering climates contribute to optimal or compromised functioning. Therefore, cluster analysis emerges as a valuable method for understanding these complex social dynamics and their impact on young athletes’ sporting experiences.

Unlike variable-centered techniques, which primarily assess interactions between specific variables, cluster analysis adopts a person-centered perspective that identifies naturally occurring profiles based on selected characteristics [see Aldenderfer and Blashfield (1984) and Hair et al. (1998) for more on cluster analysis]. As Smith et al. (2006) note, this method is particularly advantageous for uncovering “naturally occurring constellations” of social relationships in sport, allowing researchers to move beyond researcher-defined groups and focus on profiles that emerge organically from the data. Additionally, as noted by Morbée et al. (2023), in real life, social agents, often combine different interpersonal styles, leading to a combination of both adaptive and maladaptive behaviors, making this methodology particularly suitable for studying such dynamics. This approach has been widely applied in youth sport research to identify distinct psychological profiles based on athletes’ perceptions of social environments, helping explain individual differences in motivation and behavior (e.g., Harwood et al., 2004; Raedeke, 1997; Smith et al., 2006).

Based on the aforementioned, the overall aim of this study was (a) to describe the perceived motivational climate profiles created by coaches, parents, and peers of young football players, and (b) to analyse the implications of these profiles on goal orientations, motivation, basic psychological needs, and well- and ill-being variables.

Drawing from the empowering and disempowering motivational climates model (Duda, 2013; Duda et al., 2024), we expect to identify at least two profiles: an adaptive profile with high levels of empowering climate and low levels of disempowering climate, and a contrasting maladaptive profile with low levels of empowering climate and high levels of disempowering climate. In this regard, it is expected that the profile characterized by high levels of empowering climates (created by coaches, parents, and peers) will exhibit the most optimal functioning, while the profile characterized by high levels of disempowering climates will display the most compromised functioning. To frame this hypothesis, Balaguer’s (2023) conceptualization of optimal and compromised functioning in sports was employed. According to this approach, optimal functioning encompasses the satisfaction of basic psychological needs, task orientation, autonomous motivation, and well-being. Conversely, compromised functioning involves the frustration of basic psychological needs, ego orientation, controlled motivation or amotivation, and ill-being.

## 2 Methodology

### 2.1 Participants

A total of 876 youth soccer players (677 boys, 191 girls, and 8 non-binary participants) from 55 teams were recruited for the study. The athletes ranged from 11 to 18 years old (*M* = 13.57, *SD* = 1.17) and competed in officially regulated youth football leagues organized by the regional football federation of Valencia (Spain). Prior to the data collection, a minimum of 4 weeks of interaction between the athletes and their coach was required to ensure sufficient exposure to the coach-created motivational climate. Data were collected during the early competitive phase of the season. On average, athletes trained with their coach approximately three times per week (*M* = 3.10, *SD* = 0.68).

### 2.2 Procedure

The research was approved by the Human Research Ethics Committee of the university (approval number: 023-MAG-2683518). For participant selection, a random sampling was conducted from the teams in Valencia (Spain), assuming a sampling error of 0.04. Both youth football teams and social schools, as well as elite academies affiliated with professional football clubs and profit-oriented performance-specific schools, were included.

Once the teams were selected, the principal researchers of the project contacted the sports directors of each school to present the research and request their collaboration. Upon obtaining their agreement, the researchers coordinated with the sports school to set dates for administering the questionnaires. Prior to the scheduled dates, voluntary cooperation was sought from the families or legal guardians to enable the players’ participation. Specifically, they were informed about the study’s objectives and provided with an informed consent form that needed to be completed before the distribution of the questionnaires to the players.

Data collection was conducted by trained researchers at the sports facilities of the different football schools.

### 2.3 Instruments

#### 2.3.1 Perceived motivational climate created by the coach

To assess athletes’ perceptions of the motivational climate created by their coach, the Reduced Version of the Coach-created Empowering and Disempowering Motivational Climate Questionnaire (EDMCQC-R) (Atienza-González et al., 2024) was used. This is a recently developed version of the Coach-created Empowering and Disempowering Motivational Climate Questionnaire (EDMCQ-C) (Appleton et al., 2016; Appleton et al., 2023). The aim of this new version was to shorten the instrument and create a form whose content could be adapted for versions assessing the motivational climate from parents and peers. The EDMCQC-R consists of 15 items, with 9 items assessing the empowering climate dimension (e.g., “My coach could really be counted on to care, no matter what happened in soccer”) and 6 items assessing the disempowering climate dimension (e.g., “My coach yelled at players for messing up”). Participants were asked to rate the extent to which the described behaviors had been present in their soccer team over the past 3–4 weeks, using a 5-point Likert scale from 1 (*strongly disagree*) to 5 (*strongly agree*).

#### 2.3.2 Perceived motivational climate created by parents

The motivational climate created by parents was measured using the Empowering and Disempowering Motivational Climate Questionnaire - Father/Mother (EDMCQ-F/M) (Martínez-González et al., 2024). This questionnaire was developed from the Reduced Version of the Coach-created Empowering and Disempowering Motivational Climate (EDMCQC-R) (Atienza-González et al., 2024). It includes 9 items to assess the empowering climate (e.g., “My father/mother could really be counted on to care, no matter what happened in soccer”) and 6 items for the disempowering climate (e.g., “My father/mother yelled at me for messing up in soccer”), with separate versions for fathers and mothers (and adaptations for other family models). Athletes were required to respond, using a 5-point Likert scale from 1 (*strongly disagree*) to 5 (*strongly agree*), to what extent the items represented the behaviors of their father or mother during the past 3–4 weeks. Preliminary reliability and validity analyses indicated that the instrument is appropriate and valid for use either separately (father version or mother version) or jointly (parents’ version) [see Martínez-González et al. (2024)]. In this study, they were used together, so that scores were obtained for the parents’ empowering and disempowering climate.

#### 2.3.3 Perceived motivational climate created by peers

The Empowering and Disempowering Motivational Climate Questionnaire for Peers (EDMCQP) (González-García et al., 2024) was used to evaluate athletes’ perceptions of the motivational climate created by their peers. As well as the previous questionnaire, it was developed from the Reduced Version of the Coach-created Empowering and Disempowering Motivational Climate Questionnaire (EDMCQC-R) (Atienza-González et al., 2024). This instrument also consists of 15 items, with 9 items evaluating the empowering climate (e.g., “Most players always supported each other, no matter what happened in soccer”) and 6 assessing the disempowering climate (e.g., “Most players yelled at their teammates for messing up”). Athletes rated the extent to which the behaviors reflected what their teammates did or said over the past 3–4 weeks, using a scale from 1 (*strongly disagree*) to 5 (*strongly agree*).

#### 2.3.4 Goal orientations

The Spanish version of the Task and Ego Orientation in Sport Questionnaire (TEOSQ) (Duda, 1989; Duda and Nicholls, 1992), adapted by Balaguer et al. (1996), was used to assess players’ achievement goal orientations. This questionnaire comprises 13 items: 7 focused on task orientation (e.g., “I feel successful in sport when I work really hard”) and 6 assessing ego orientation (e.g., “I feel successful in sport when others cannot perform as well as I do”). Participants respond using a 5-point Likert scale, where 1 indicates strong disagreement and 5 indicates strong agreement. Previous research with Spanish athletes has shown that this scale has satisfactory reliability (*α* = 0.76 to 0.88) and validity (Balaguer et al., 2011a; Castillo et al., 2002; Castillo et al., 2010).

#### 2.3.5 Basic psychological needs

The assessment of athletes’ basic psychological needs satisfaction was conducted using an instrument created specifically for this research. The questionnaire was developed based on Spanish adaptations (Balaguer et al., 2008) of the following items or subscales to asses competence, autonomy, and relatedness, respectively: (a) the competence subscale from the Intrinsic Motivation Inventory (IMI) (McAuley et al., 1989); (b) 5 items to measure autonomy proposed by Standage et al. (2005); and (c) the acceptance subscale from the Need for Relatedness Scale (NRS) (Richer and Vallerand, 1998). The final questionnaire contained 10 items, of which four items assessed the need for competence (e.g., “I am satisfied with what I have done”), four assessed the need for autonomy (e.g., “I have felt the freedom to do some things my own way”), and three assessed the need for relatedness (e.g., “I have felt that people understood me”).

In addition, basic psychological needs frustration was assessed using an *ad hoc* short version of the Spanish adaptation (Balaguer et al., 2010) of the Psychological Need Thwarting Scale (PNTS) (Bartholomew et al., 2011). The short version consisted of 9 items, which assessed the need for competence (3 items, e.g., “I feel inadequate because I am not given opportunities to fulfill my potential”), the need for autonomy (3 items, e.g., “I feel pushed to behave in certain ways”), and the need for relatedness (3 items, e.g., “I feel I am rejected by those around me”).

Both the basic psychological needs satisfaction scale and the basic psychological needs frustration scale were answered using a Likert-type response scale from 1 (*strongly disagree*) to 5 (*strongly agree*).

#### 2.3.6 Motivation

Athletes’ motivational regulations were assessed using a short version of the Spanish adaptation of the Behavioral Regulation in Sport Questionnaire (BRSQ) (Lonsdale et al., 2008; Viladrich et al., 2013). The short questionnaire consists of 13 items, which can be grouped into autonomous motivation (5 items, e.g., “I play my sport because I enjoy it”), controlled motivation (4 items, e.g., “I play my sport because people push me to play”), and amotivation (4 items, e.g., “I play my sport, but I question why I continue”). Responses were collected on a Likert scale ranging from 1 (*strongly disagree*) to 5 (*strongly agree*).

#### 2.3.7 Well- and ill-being

As an indicator of well-being, athletes’ subjective feelings of energy and vitality were assessed using the Spanish version (Castillo et al., 2017) of the Subjective Vitality Scale (Ryan and Frederick, 1997). The scale consists of 6 items (e.g., “I feel alive and vital”), rated on a 7-point Likert scale from 1 (*not at all true*) to 7 (*very true*). This is a well-established instrument that has demonstrated high reliability and validity (*α* = 0.77 to 0.89) in the sports context (Álvarez et al., 2012; Balaguer et al., 2012; Balaguer et al., 2018; González et al., 2015; Martínez-González et al., 2021).

Regarding ill-being, athletes’ physical and emotional exhaustion was assessed. For this purpose, the corresponding subscale from the Spanish version (Balaguer et al., 2011b) of the Athlete Burnout Questionnaire (ABQ) (Raedeke and Smith, 2001) was employed. This subscale consists of 5 items (e.g., “I am exhausted by the mental and physical demands of soccer”), which are rated on a Likert scale ranging from 1 (*almost never*) a 5 (*almost always*). This subscale has demonstrated both adequate validity and reliability (*α* = 0.76 to 0.89) as an indicator of ill-being in previous studies within the sports context (Adie et al., 2008; Castillo et al., 2010; Madigan and Nicholls, 2017; Martínez-González et al., 2021).

### 2.4 Data analysis

Descriptive statistics, reliability, bivariate correlations, and the between-subjects multivariate analysis of covariance (MANCOVA) were analyzed using IBM SPSS Statistics 25 software. In addition, MPlus Version 7 (Muthén and Muthén, 2012) was used to carry out confirmatory factor analyses, while Weka 3.8.6 software (Hall et al., 2009) was employed for cluster analyses.

For confirmatory factor analyses, the following goodness-of-fit indices were used: chi-square (*χ*^2^), comparative fit index (CFI), Tucker-Lewis index (TLI), standardized root mean square residual (SRMR), and root mean square error of approximation (RMSEA). CFI and TLI values greater than 0.90 indicate an acceptable fit (Hu and Bentler, 1995). RMSEA and SRMR values between 0.05 and 0.10 are considered acceptable, with values below 0.08 being considered optimal (Cole and Maxwell, 1985).

Regarding cluster analyses, the SimpleKMeans algorithm, which is included by default in Weka, was employed. This algorithm was selected due to its capacity to partition the data into distinct clusters based on similarity measures (see Arthur and Vassilvitskii, 2007).

Finally, for the MANCOVA, assumptions regarding normality, homogeneity of variance–covariance matrices, linearity, and multicollinearity were tested prior to conducting the main analyses. Partial *η*^2^ was calculated to estimate the effect sizes, with Cohen (1988) guidelines used for interpretation. Specifically, values of 0.01, 0.06, and 0.14 were regarded as indicative of small, medium, and large effects, respectively.

## 3 Results

### 3.1 Preliminary analyses

Regarding CFA, results for all motivational climate scales revealed adequate fit to the data: coach motivational climate [χ^2^(87) = 284.69, *p* < 0.01; CFI = 0.97; TLI = 0.97; RMSEA = 0.052], and peer motivational climate [χ^2^(87) = 303.37, *p* < 0.01; CFI = 0.98; TLI = 0.98; RMSEA = 0.054]. In the case of the parental motivational climate, two items from the disempowering climate scale (My father/mother had his or her favorite players; My father/mother thought that only the best players should play in a match) had to be removed due to issues with reliability and validity. After their removal, the confirmatory factor analyses were found to be acceptable: [χ^2^(59) = 1479.69, *p* < 0.01; CFI = 0.96; TLI = 0.95; RMSEA = 0.071].

The fit indices were also satisfactory for task and ego orientation [χ^2^(64) = 314.94, *p* < 0.01; CFI = 0.96; TLI = 0.95; RMSEA = 0.067], motivation [χ^2^(62) = 314.72, *p* < 0.01; CFI = 0.98; TLI = 0.97; RMSEA = 0.068], basic psychological need satisfaction [χ^2^(31) = 162.92, *p* < 0.01; CFI = 0.99; TLI = 0.98; RMSEA = 0.070], basic psychological need frustration [χ^2^(24) = 156.64, *p* < 0.01; CFI = 0.99; TLI = 0.98; RMSEA = 0.079], vitality [χ^2^(7) = 40.02, *p* < 0.01; CFI = 0.99; TLI = 0.99; RMSEA = 0.073], and physical and emotional exhaustion [χ^2^(4) = 11.44, *p* < 0.01; CFI = 0.99; TLI = 0.99; RMSEA = 0.046].

### 3.2 Descriptive statistics, reliability and bivariate correlations

The descriptive statistics, internal consistency, and bivariate correlations for the variables are presented in Table 1. Cronbach’s alpha coefficients were satisfactory for all variables, ranging from 0.71 to 0.89. Athletes’ means revealed high empowering climates and low disempowering climates across coaches, parents, and peers. Additionally, high scores were observed in adaptive variables (task orientation, autonomous motivation, needs satisfaction, vitality) and low scores in maladaptive variables (ego orientation, controlled motivation, amotivation, needs frustration, exhaustion).

**Table 1:** Descriptive statistics, reliabilities, and bivariate correlations.

Variables	*M*	*SD*	*α*	1	2	3	4	5	6	7	8	9	10	11	12	13	14
1 Coach empowering climate	4.26	0.52	0.82	-													
2 Coach disempowering climate	2.53	0.73	0.71	−0.47^**^	-												
3 Parental empowering climate	4.45	0.47	0.80	0.43^**^	−0.16^**^	-											
4 Parental disempowering climate	2.05	0.64	0.82	−0.24^**^	0.38^**^	−0.32^**^	-										
5 Peer empowering climate	4.05	0.64	0.88	0.55^**^	−0.27^**^	0.43^**^	−0.16^**^	-									
6 Peer disempowering climate	2.78	0.75	0.73	−0.31^**^	0.54^**^	−0.19^**^	0.38^**^	−0.42^**^	-								
7 Task orientation	4.37	0.50	0.78	0.35^**^	−0.06	0.36^**^	−0.16^**^	0.27^**^	−0.03	-							
8 Ego orientation	3.03	0.80	0.81	−0.13^**^	0.23^**^	−0.08^*^	0.17^**^	−0.05	0.24^**^	0.13^**^	-						
9 Autonomous motivation	4.55	0.52	0.75	0.39^**^	−0.16^**^	0.39^**^	−0.17^**^	0.39^**^	−0.14^**^	0.34^**^	−0.02	-					
10 Controlled motivation	1.93	0.90	0.74	−0.28^**^	0.32^**^	−0.25^**^	0.38^**^	−0.26^**^	0.32^**^	−0.13^**^	0.18^**^	−0.24^**^	-				
11 Amotivation	1.74	0.86	0.80	−0.34^**^	0.29^**^	−0.34^**^	0.34^**^	−0.23^**^	0.30^**^	−0.19^**^	0.15^**^	−0.41^**^	0.59^**^	-			
12 BPNS	3.99	0.59	0.85	0.43^**^	−0.18^**^	0.35^**^	−0.08^*^	0.50^**^	−0.18^**^	0.29^**^	0.02	0.47^**^	−0.17^**^	−0.24^**^	-		
13 BPNF	2.21	0.80	0.89	−0.38^**^	0.43^**^	−0.31^**^	0.29^**^	−0.44^**^	0.46^**^	−0.15^**^	0.12^**^	−0.31^**^	0.45^**^	0.48^**^	−0.48^**^	-	
14 Vitality	4.05	0.75	0.89	0.33^**^	−0.14^**^	0.33^**^	−0.09^*^	0.29^**^	−0.15^**^	0.22^**^	−0.01	0.37^**^	−0.20^**^	−0.24^**^	0.55^**^	−0.34^**^	-
15 Physical and emotional exhaustion	1.74	0.75	0.84	−0.23^**^	0.21^**^	−0.20^**^	0.23^**^	−0.25^**^	0.23^**^	−0.18^**^	−0.05	−0.22^**^	0.34^**^	0.34^**^	−0.24^**^	0.42^**^	−0.28^**^

^*^p < 0.05; ^**^p < 0.01. BPNS, basic psychological needs satisfaction; BPNF, basic psychological needs frustration.

With respect to the relationships between variables, all relationships aligned with theoretical frameworks and previous literature. Specifically, empowering climates created by coaches, parents, and peers were positively interrelated, indicating that empowering behaviors across different significant others are consistent. Similarly, disempowering climates from coaches, parents, and peers showed significant positive correlations among them.

Significant relationships were also observed between empowering climates and positive outcomes. For instance, empowering climates created by coaches, parents, and peers were significantly and positively associated with task orientation, autonomous motivation, athletes’ needs satisfaction, and vitality, reflecting their role in fostering adaptive experiences.

Conversely, disempowering climates exhibited significant positive associations with ego orientation, controlled motivation, amotivation, needs frustration, and physical and emotional exhaustion, reinforcing their connection to maladaptive outcomes.

### 3.3 Cluster analyses

**Figure 1:**
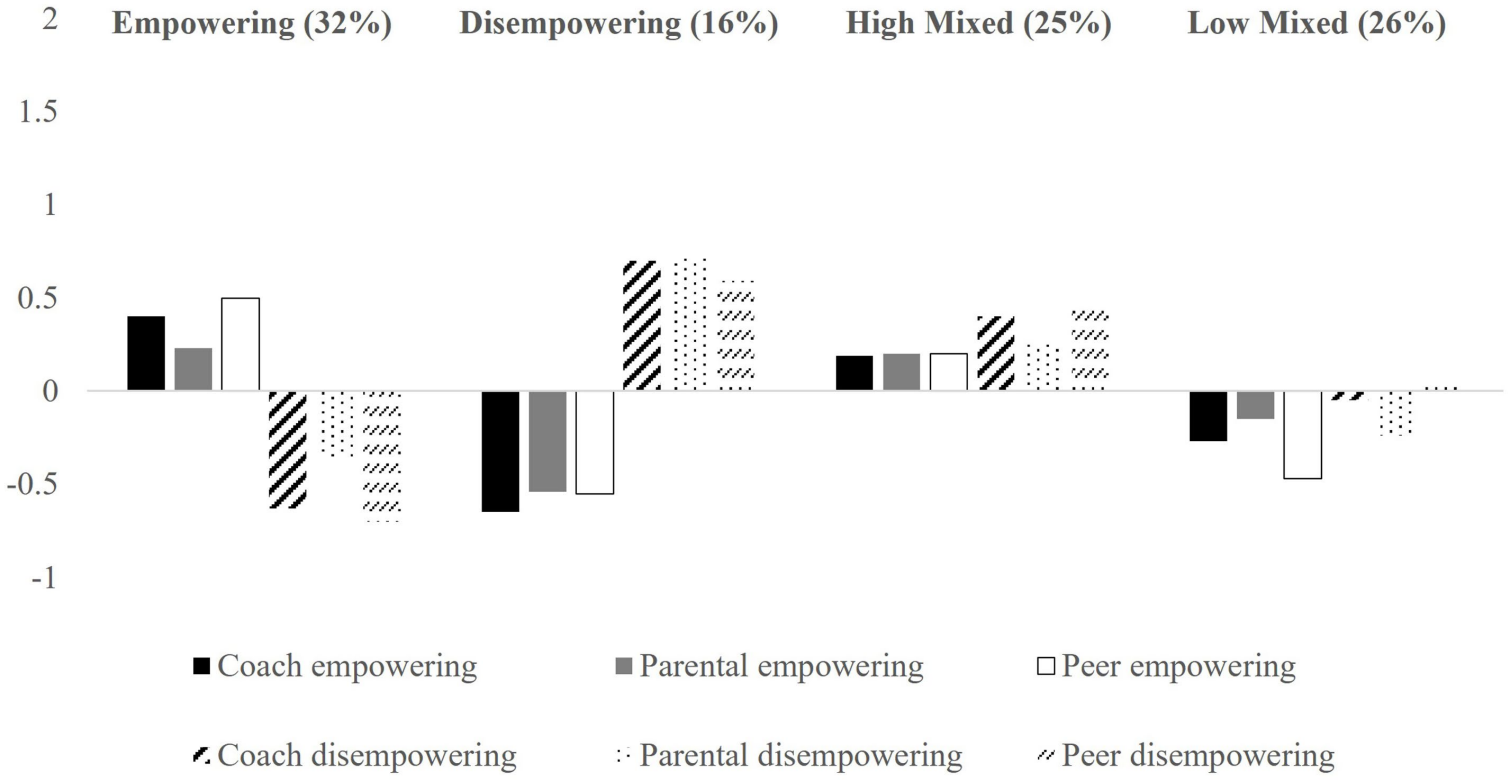
Results of *k*-means cluster analysis (*N* = 876).

The analysis was conducted using the Euclidean distance metric and involved a total of 876 instances across 6 attributes (empowering and disempowering motivational climate of coach, parents and peers). Results of cluster analysis revealed four distinct motivational profiles (see Figure 1) that best fit the data, providing a comprehensive solution for understanding the varying perceptions of empowering and disempowering climates created by significant others.

Two of these profiles aligned with the typical models of empowering and disempowering climates. At one extreme, it is the most represented cluster (32%), labelled “Empowering,” which exhibited high levels of empowering climate and low levels of disempowering climate. At the other extreme, the least represented cluster (16%), labelled “Disempowering,” was characterized by low levels of empowering climate and high levels of disempowering climate.

Additionally, two more clusters emerged. The cluster labelled “High Mixed,” displayed moderated levels of both empowering climates and disempowering climates. This suggests a complex, mixed experience where athletes perceive both empowering and disempowering behaviors in their environment. Finally, the “Low Mixed” cluster revealed low levels of both empowering and disempowering climates.

### 3.4 MANCOVA

A multivariate analysis of covariance (MANCOVA) was conducted to evaluate the effect of cluster membership on various optimal functioning variables while controlling for gender and age (see Table 2). The dependent variables included task and ego orientation, autonomous motivation, controlled motivation, and amotivation, need satisfaction and need frustration, vitality, and physical emotional exhaustion. The independent variable was cluster membership, with four clusters identified. Gender and age were used as covariates.

**Table 2:** Cluster means, standard errors, and analyses of variance in optimal functioning-related variables.

Measure	Empowering (*n* = 283)		Disempowering (*n* = 140)		High mixed (*n* = 222)		Low mixed (*n* = 231)		*F*(3, 870)	*η* ^2^
	*M*	*SD*	*M*	*SD*	*M*	*SD*	*M*	*SD*		
Task orientation	4.50	0.03	4.09	0.04	4.52	0.03	4.24	0.03	38.51^**^	0.117
Ego orientation	2.79	0.05	3.22	0.07	3.21	0.05	3.02	0.05	15.83^**^	0.052
Autonomous motivation	4.76	0.34	4.19	0.60	4.66	0.42	4.40	0.59	54.94^**^	0.159
Controlled motivation	1.56	0.69	2.67	0.96	1.99	0.94	1.90	0.77	54.27^**^	0.158
Amotivation	1.38	0.60	2.52	0.97	1.70	0.84	1.75	0.78	67.86^**^	0.190
BPNS	4.25	0.50	3.65	0.57	4.12	0.60	3.74	0.52	60.85^**^	0.173
BPNF	1.69	0.58	2.83	0.74	2.30	0.77	2.38	0.73	93.29^**^	0.243
Vitality	4.33	0.71	3.71	0.68	4.15	0.73	3.81	0.73	35.78^**^	0.110
Physical and emotional exhaustion	1.44	0.56	2.11	0.90	1.79	0.78	1.83	0.70	30.34^**^	0.095

^**^p < 0.01. BPNS, basic psychological needs satisfaction; BPNF, basic psychological needs frustration.

The combined DVs were significantly different by cluster [Pillai’s Trace = 0.50, *F* (27, 2592) = 19.15, *p* < 0.001, *η_p_*^2^ = 0.166], after controlling for gender and age. This suggests that approximately 16.6% of the variance in the dependent variables is accounted for by the cluster differences, indicating a moderate to large effect size.

Gender also showed a significant multivariate effect [Pillai’s Trace = 0.040, *F* (9, 862) = 3.98, *p* < 0.001, *η_p_*^2^ = 0.040], indicating that gender explains about 4.0% of the variance in the dependent variables. Specifically, gender was a significant covariate for subjective vitality [*F* (1, 870) = 12.35, *p* < 0.001, *η_p_*^2^ = 0.014].^1^

Finally, age also had a significant multivariate effect [Pillai’s Trace = 0.055, *F* (9, 862) = 5.60, *p* < 0.001, *η_p_*^2^ = 0.055], suggesting that age contribute significantly to 5.5% of the variance. Specifically, age was a significant covariate for ego orientation [*F* (1, 870) = 23.32, *p* < 0.001, *η_p_*^2^ = 0.026] and subjective vitality [*F* (1, 870) = 17.58, *p* < 0.001, *η_p_*^2^ = 0.020].^2^

The between-subjects effects for each dependent variable indicated that cluster membership significantly influenced all studied variables. The most pronounced effects were observed for need frustration (*η_p_*^2^ = 0.243), and need satisfaction (*η_p_*^2^ = 0.173), as well as for the three forms of motivation (*η_p_*^2^ for autonomous motivation = 0.159; *η_p_*^2^ for controlled motivation = 0.158; *η_p_*^2^ for amotivation = 0.190), indicating that cluster differences are particularly influential in these variables.

Pair-wise comparison revealed significant differences between clusters in the means of the analysed variables, highlighting their unique psychological profiles. Empowering profile consistently stood out with the highest levels of autonomous motivation, need satisfaction, and vitality, along with the lowest levels of ego orientation, controlled motivation, amotivation, need frustration, and physical and emotional exhaustion. This cluster appears to represent the most optimal functioning group. In contrast, disempowering profile exhibited the most concerning cluster, with the highest levels of controlled motivation, amotivation, need frustration, and physical and emotional exhaustion, alongside the lowest scores in autonomous motivation, need satisfaction, and vitality. This suggests that individuals in this cluster experience the most compromised functioning.

The remaining clusters fell between these extremes. High Mixed profile showed high task orientation, high autonomous motivation and need satisfaction, similar to Empowering profile, but with slightly higher controlled motivation and need frustration. Finally, Low Mixed profile, is more balanced with moderate scores across all variables, indicating a mix of both positive and negative outcomes (e.g., needs satisfaction; needs frustration).

## 4 Discussion

Within the framework of the integrated model of empowering and disempowering climate (Duda, 2013; Duda et al., 2024), the aim of this study was (a) to describe the perceived motivational climate profiles created by coaches, parents, and peers of young football players, and (b) to analyse the implications of these profiles on goal orientations, motivation, psychological needs, and well- and ill-being variables.

It was hypothesized that at least two contrasting profiles would emerge: one characterized by high empowering and low disempowering climates, associated with optimal functioning; and another showing low empowering and high disempowering climates, related to compromised functioning. Following Balaguer (2023) conceptualization, optimal functioning indicators included task orientation, satisfaction of basic psychological needs, autonomous motivation, and vitality, whereas compromised functioning encompassed ego orientation, frustration of basic psychological needs, controlled motivation or amotivation, and physical and emotional exhaustion.

The sample consisted of youth soccer players, representing the most widely practiced organized sport in our country, spanning the developmental stages of childhood and adolescence. These stages are marked by the continued significance of parents’ roles and the increasing influence of peers (Fredricks and Eccles, 2004; Harwood and Knight, 2015; Le Bars et al., 2009; O’Rourke et al., 2014). The analysis identified four distinct profiles among the soccer players in the study, characterized by varying levels of perceived empowering and disempowering climates.

The most represented profile was the “empowering” group, comprising athletes who perceived that their coaches, parents, and peers significantly contributed to creating a social environment that fostered feelings of competence, autonomy, and relatedness. This environment also promoted effort, learning, and task mastery. It was the climate with the least perceived coercive or authoritarian behaviors and the lowest emphasis on ego-involvement.

At the other extreme was the “disempowering” profile, which was the least represented among the participants. This profile included athletes who perceived that their coaches, parents, and peers exhibited controlling behaviors, imposed ways of thinking, feeling, and behaving, and emphasized judging competence based on normative criteria. These behaviors fostered social comparison, rivalry, and an emphasis on outcomes beyond their control, with mistakes being punished and demonstrations of ability and superiority being reinforced. This was also the climate with the lowest levels of perceived autonomy-supportive behaviors and the least encouragement of task mastery.

Together, these two opposite profiles accounted for almost half of the sample analysed. The other half of the sample was represented by two mixed profiles, termed “high mixed” and “low mixed.” These profiles reflected climates without a marked distinction between empowering and disempowering characteristics or, in other words, where social agents exhibited a varied repertoire of behaviors without a clear inclination. The “high mixed” profile reflected a climate where coaches, parents, and peers exhibited moderate levels of empowering behaviors. While adaptive behaviors typical of the “empowering” profile were present—such as autonomy-supportive behaviors and task-involvement—they were not as pronounced as in the fully empowering profile. Simultaneously, social agents also demonstrated moderate-to-high levels of disempowering behaviors, including controlling behaviors and an emphasis on ego-involvement. Finally, the “low mixed” profile was characterized by low levels of both empowering and disempowering climates, suggesting limited involvement of social agents in the athletes’ sports environment.

### 4.1 Differences in optimal functioning across groups

The most substantial differences were found in the quality of motivation and the extent to which athletes’ basic psychological needs for competence, autonomy, and relatedness were satisfied or frustrated by their sports environment.

Supporting our hypotheses and in line with the theoretical framework, athletes in the “empowering” group exhibited the highest levels of optimal functioning. They reported elevated task orientation, the highest levels of autonomous motivation and satisfaction of basic psychological needs, and greater vitality compared to the other groups. They also showed the lowest levels of ego orientation, controlled motivation and amotivation, frustration of basic psychological needs, and physical and emotional exhaustion. These athletes thus enjoyed a high-quality sports experience.

On the other hand, athletes in the “disempowering” group displayed the most compromised functioning. They exhibited higher ego orientation, the highest levels of controlled motivation and amotivation, and greater frustration of their basic psychological needs and physical and emotional exhaustion. Their pursuit of mastery was the lowest among all groups, as were their levels of autonomous motivation and vitality.

Although no previous studies have examined coaches’, parents’, and peers’ motivational climates from this theoretical perspective using a person-centered approach, these findings are consistent with the theory (Duda, 2013; Duda et al., 2024). Additionally, results align with prior variable-centered studies that have positively associated empowering motivational climates with task orientation (Ruiz et al., 2021), autonomous motivation (Fenton et al., 2017; Mosqueda et al., 2019; Ruiz et al., 2021), satisfaction of basic psychological needs (Chu et al., 2021; Hancox et al., 2017; Mosqueda et al., 2022), and well-being indicators (Appleton and Duda, 2016). In contrast, disempowering motivational climates have been linked to poorer quality motivation and ego orientation (Ruiz et al., 2021), frustration of basic psychological needs (Chu et al., 2021; Mosqueda et al., 2022), and increased physical and psychological ill-being (Hancox et al., 2017; Into et al., 2020).

Regarding mixed profiles, in the “high mixed” profile, athletes reported relatively high scores on all adaptive variables but also elevated maladaptive indicators, suggesting that simultaneous exposure to empowering and disempowering behaviors from coaches, parents, and peers limits the benefits of empowering climates. In contrast, the “low mixed” profile reflected low involvement from social agents, with athletes reporting below-average adaptive scores and slightly elevated need frustration and exhaustion, indicating an unsatisfactory sporting experience marked by ambivalence between adaptive and maladaptive feelings. These findings highlight that a combination of high empowering and disempowering behaviors—or low levels of both—can undermine the full potential of empowering climates for young athletes’ optimal functioning.

Similar results were reported by Morbée et al. (2023) in a recent study of young athletes. Their analysis of parental profiles related to the satisfaction or frustration of athletes’ basic psychological needs identified four profiles: a “need-supportive” profile, a “need-thwarting” profile, a “predominantly controlling” profile, and a “distant” profile characterized by low levels of all perceived behaviors.

### 4.2 Theoretical and practical implications

These findings have significant theoretical and practical implications. Among the theoretical implications, this study is the first to analyze, from a person-centered perspective, the motivational profiles created by coaches, parents, and peers, providing empirical support for the integrated model of empowering and disempowering motivational climates (Duda, 2013; Duda et al., 2024). The results highlight that significant others can simultaneously exhibit empowering and disempowering behaviors, underscoring the need to evaluate both dimensions to fully understand the motivational climate. The “high mixed” and “low mixed” profiles demonstrate that either the coexistence of empowering and disempowering behaviors or low involvement limits the benefits of empowering climates for optimal athlete functioning. This connects with one of the most important practical implications of this research: it offers a framework to identify and address less adaptive profiles to improve young athletes’ sports experiences and promote their well-being. Based on our data, we can infer that only about one-third of the young soccer players who participated in this study are benefiting from adaptive motivational climates in their sports environment. Identifying these profiles is crucial for enhancing their optimal functioning in the terms discussed throughout this work. Therefore, interventions should focus on guiding coaches, parents, and peers to reduce disempowering behaviors while fostering empowering climates to maximize athletes’ functioning.

Finally, it is important to highlight another strength of this article: the use of recently validated instruments that assess motivational climates created by coaches, parents, and peers from a common perspective. This addresses a limitation faced by professionals in the sports field, as they previously had to work with instruments that were not analogous for all involved social agents and did not assess motivational climates in the same terms. Thanks to these new instruments, professionals will be able to benefit from and evaluate the motivational climates perceived by athletes, created by their coaches, parents, and peers, and intervene accordingly.

### 4.3 Limitations and future directions

Despite these strengths, this study has several limitations that should be acknowledged. First, the study employs a cross-sectional design, enabling the analysis of profiles at a specific moment in time. However, future research is encouraged to examine these profiles longitudinally. Unlike Latent Profile Analysis (LPA), where latent classes represent stable sets of characteristics, techniques such as Latent Profile Transition Analysis (LPTA) allows for individuals to change membership in latent classes over time (Lanza et al., 2010). Therefore, the application of LPTA would allow for the analysis of the stability or changes in profiles over time, as some studies have already been done in the sports context (see Martinent and Decret, 2015).

Second, due to the research design, this study does not address explanatory mechanisms. While the results confirm which motivational climate profiles created by significant others are associated with optimal functioning and which with compromised functioning, the relationships between variables leading to these consequences are not tested. Although the study includes variables from all levels of the hierarchical model of empowering and disempowering climates, it does not test the relationships as other studies within this theoretical framework have done (see Castillo-Jiménez et al., 2022; Krommidas et al., 2022; Martínez-González et al., 2021; Mosqueda et al., 2022; Ruiz et al., 2021).

Third, it is important to note that one of the evaluation scales requires further exploration. This refers to the parental disempowering climate scale, where two items had to be removed due to issues with reliability and validity. These items reflected behaviors typical of ego-involving climates, specifically unequal treatment based on skill levels. Although the result is a valid and reliable instrument that can be used in different versions (father version, mother version, and parents’ version), it should be noted that, in the case of parents, the instrument does not capture the full range of behaviors characteristic of a disempowering climate. This is particularly relevant for studies aiming to jointly use the three motivational climate instruments (coach, parent, and peer) and analyze their comparative effects or interactions. Although additional analyses were conducted, including comparisons between the adjusted parental scale and the complete versions of the other scales, which supported its suitability, future research should continue to explore the functioning of these items and follow the authors’ recommendations for their application [see Martínez-González et al. (2024)].

Finally, it may be valuable to complement athletes’ perceptions with other measures of motivational climates. For instance, the inclusion of direct and objective measures, such as observed motivational climates, could prove beneficial. To date, within the theoretical framework of reference, only the Multidimensional Motivational Climate Observation System (MMCOS; Smith et al., 2015) has been developed to assess the empowering and disempowering motivational climates created by coaches. Future studies could adapt this instrument to evaluate, through an observation system, the behaviors of parents or peers in the sports context. While discrepancies may exist between real and perceived behaviors, it is evident that athletes’ perceptions are critical to their sports experiences (Morbée et al., 2023), which gives meaning to this type of study. Additionally, other aspects such as gender and cultural context, which were not specifically analyzed in this study, should be explored in future research to determine whether motivational climate profiles and their associations with functioning vary across genders and cultural contexts.

## 5 Conclusion

In summary, this study offers valuable insights into the motivational climate profiles created by coaches, parents, and peers within the framework of the integrated model of empowering and disempowering climates (Duda, 2013; Duda et al., 2024). By employing a person-centered approach, we identified four distinct profiles that varied in their combination of empowering and disempowering levels: “empowering,” “disempowering,” “high mixed,” and “low mixed.” These profiles highlight the critical role of significant social agents in shaping young athletes’ functioning in sports.

Notably, athletes who perceive that their coaches, parents, and peers create motivational climates characterized by high empowering and low disempowering behaviors benefit most from optimal functioning, while those who perceive high levels of disempowering and low levels of empowering behaviors experience the most compromised functioning in their sports practice. Mixed profiles reveal that combining empowering and disempowering behaviors limits the benefits of empowering climates, and low involvement hinders athletes’ development. These findings emphasize the need for targeted interventions to promote empowering behaviors and minimize disempowering influences in youth sports.

## Data Availability

The raw data supporting the conclusions of this article will be made available by the authors, without undue reservation.

## Footnotes

^1^In this research, analyses revealed that boys score significantly higher than girls in subjective vitality.

^2^In this research, analyses revealed that cadet football players scored significantly higher in ego orientation and lower in vitality compared to junior players.
